# LIVING WELL WITH MILD TO MODERATE DEMENTIA. FINDINGS FROM THE IDEAL PROGRAM: A BRITISH COHORT STUDY

**DOI:** 10.1093/geroni/igad104.1428

**Published:** 2023-12-21

**Authors:** Linda Clare, Christina Victor

## Abstract

IDEAL (Improving the Experience of Dementia and Enhancing Active Life) is a British-based cohort study that commenced in 2014 and recruited 1545 people with dementia and 1277 carers, following them for 3 waves at yearly intervals. IDEAL-2 continued to follow those who remained in the study for a further 3 waves and incorporated 205 additional participants as part of an enrichment cohort. A key research and policy goal is to understand the factors that promote or inhibit ‘living well’ for people with dementia and their carers, defined as the best achievable state of physical, mental and social health. In this symposium we examine how living well outcomes vary longitudinally and with social (minority ethnic status) factors. Two papers focus on quality of life of people with dementia longitudinally. Our first paper shows that for most study participants quality of life scores remained stable. For those with declining quality of life key associated factors include demographic characteristics, cognition, and psychological health including depression. Our second paper has a clinical dimension and demonstrates that, beyond the point of diagnosis, quality of life does not vary according to dementia subtype. Our third paper focuses on social health using social isolation as our measure. Isolation increased over time and was higher in carers than people with dementia. Our final paper demonstrates that, compared with their white British counterparts, minority ethnic carers report higher stress and role captivity and lower relationship quality while people with dementia have lower quality of life and higher loneliness.

